# Invasive Hemodynamic Changes Associated With Sodium-Glucose Cotransporter-2 Inhibitors: A Systematic Review and Meta-Analysis of Randomized Controlled Trials

**DOI:** 10.1155/2024/2735577

**Published:** 2024-05-27

**Authors:** Abiodun Idowu, Olayinka Adebolu, Casipit Bruce, Bede Nriagu, Endurance Evbayekha, Kevin Bryan Lo, Olayinka Afolabi-Brown, Janani Rangaswami

**Affiliations:** ^1^ Department of Medicine Einstein Medical Center, Philadelphia, Pennsylvania, USA; ^2^ Department of Medicine New York Medical College Metropolitan Hospital Center, New York City, New York, USA; ^3^ Department of Medicine St. Luke's Hospital, Chesterfield, Missouri, USA; ^4^ Department of Cardiology Brigham and Women's Hospital, Boston, Massachusetts, USA; ^5^ Division of Nephrology George Washington University School of Medicine, Washington, DC, USA; ^6^ Division of Nephrology Veterans Affairs Medical Center, Washington, DC, USA

**Keywords:** invasive hemodynamics, mean pulmonary artery pressure, pulmonary capillary wedge pressure, right heart catheterization, sodium-glucose cotransporter-2 inhibitors

## Abstract

Sodium-glucose cotransporter-2 inhibitors (SGLT2is) have been shown to lower incident heart failure (HF) and HF hospitalizations, but the mechanisms of benefit in relation to invasive hemodynamics remain unclear. Using PRISMA guidelines, we systematically reviewed multiple online databases for randomized trials evaluating the effect of SGLT2i on invasive hemodynamics. Rest and peak exercise invasive hemodynamics were measured via right heart catheterization pre- and postintervention. Random effects model meta-analysis at a 95% confidence interval was done using RevMan 5.0. A total of 3 studies with a total of 145 patients were included in the meta-analysis. SGLT2i was significantly associated with a reduction in pulmonary capillary wedge pressure at rest and peak exercise. Similarly, SGLT2i reduced mean pulmonary artery pressure at rest and peak exercise, respectively; however, this was not statistically significant. This hypothesis-generating study offers mechanistic insights into the central hemodynamic effects of SGLT2i underpinning the HF benefits of SGLT2i.

## 1. Introduction

Sodium-glucose cotransporter-2 inhibitors (SGLT2is) have been shown to lower incident heart failure (HF), HF hospitalizations, and cardiovascular-related mortality [1–5]. Though SGLT2i promotes natriuresis and osmotic diuresis, plasma volume reduction, modest increase in ketogenesis, and decreased intraglomerular hyperfiltration, the exact mechanism by which SGLT2i mediates consistent favorable cardiorenal outcomes irrespective of patients' diabetes status and left ventricular ejection fraction (LVEF) is yet to be fully understood [6].

Congestion is a hallmark of HF which is associated with increased morbidity and mortality, and some of the benefits mediated by SGLT2i may be secondary to its diuretic effect. It however appears that these benefits may be beyond diuretic effects because, unlike SGLT2i, traditional diuretics can also induce natriuresis and diuresis without mortality benefits [7]. It has been proposed that SGLT2i reduces interstitial volume without causing significant intravascular volume depletion, which implies that SGLT2i can affect volume redistribution [8]. It however remains unclear how SGLT2i and their effects on volume status may impact central hemodynamics. Understanding the central hemodynamic effect of SGLT2i will increase our mechanistic understanding underpinning its beneficial cardiovascular effects and give an estimate of its effectiveness in reducing left ventricular filling pressures and pulmonary artery pressures. This may have a clinical implication not only for the management of HF patients but also for those with pulmonary hypertension. Therefore, this study is aimed at (a) determining changes in pulmonary capillary wedge pressure (PCWP) both at rest and exercise in patients on SGLT2i compared to those on placebo and (b) evaluating the impact of SGLT2i, compared to placebo, on rest and peak exercise mean pulmonary artery pressure.

## 2. Materials and Methods

### 2.1. Data Sources and Searches

This systematic review conformed to the Preferred Reporting Items for Systematic Review and Meta-analysis (PRISMA) protocols [9]. We queried PubMed, Embase, Ovid Medline, and Cochrane Central Register of Controlled Trials from inception to 30 August 2023 for studies that evaluated the effects of SGLT2i on central hemodynamics. The searched keywords and strategy are [(SGLT2i) OR (Sodium-Glucose Cotransporter 2 inhibitors) OR (Empagliflozin) OR (Canagliflozin) OR (Dapagliflozin) OR (Bexagliflozin) OR (Ertugliflozin) OR (Brenzavvy) OR (Invokana) OR (Farxiga) OR (Jardiance) OR (Steglatro)] AND [(hemodynamics) OR (central hemodynamics) OR (Right heart catheterization) OR (invasive hemodynamics) OR (Filling Pressures) OR (cardiac catheterization) OR (Pulmonary capillary wedge pressure) OR (Wedge pressure) OR (pulmonary artery pressure) OR (mean pulmonary artery pressure)]. We applied filter for randomized controlled trials to exclude observational and nonrandomized studies but did not apply filter to exclude studies based on publication year or language.

### 2.2. Study Selection and Data Extraction

Two reviewers (AI and OA) independently screened titles and abstracts of retrieved articles to identify potentially eligible studies. The same two investigators then read the full texts of retained citations. Discrepancies were resolved by a third reviewer (CB). The bibliographies of eligible articles were also examined to identify additional studies. The prespecified inclusion criteria were (a) human studies (randomized controlled trials, clinical trials, or prospective studies), (b) a group on SGLT2i (experimental group) and another on placebo (control group), (c) intervention duration should be at least 12 weeks, (d) invasive hemodynamics must be obtained by right heart catheterization, and (e) at least one endpoint of interest must be reported.

Two independent reviewers (BN and AI) assessed the risk of bias and extracted data from eligible studies. Risk of bias was assessed via the Revised Cochrane Risk-of-Bias Tool for Randomized Trials (RoB2) tool which has five domains (Table 1) [10]. We used the RoB2 tool algorithm (available online at http://www.riskofbias.com) that maps responses to signaling questions to reach a judgment of either “low risk,” “unclear risk,” or “high risk.” When there are some concerns but no detailed information to reliably conclude, the risk is judged as “unclear” [10]. Extracted data included authors' name, publication year, country of study, sample size, study design, patient's baseline characteristics, dosage, duration of SGLT2i, and reports of measure of effects or data needed to estimate the measure of effect from the included studies.

### 2.3. Study Endpoints

The primary endpoint of interest was a change in PCWP at rest and peak exercise (cycle ergometer exercise) pre- and postintervention. The secondary endpoint of interest was the change in mean pulmonary artery pressures. Included patients were on guideline-directed medical therapy, and SGLT2i was added in the experimental group compared to placebo for 12–24 weeks. Rest and peak exercise central hemodynamics were measured via right heart catheterization pre- and postintervention.

### 2.4. Data Analysis

The quantitative analysis for the study endpoints was done by pooling the mean and standard error of mean (SE). An inverse-variance weighting and random effects model meta-analysis was performed for the included studies in line with the DerSimonian and Laird method in anticipation of the heterogeneity of the eligible studies [11]. Higgins's *I*^2^ test was used to determine heterogeneity, with an *I*^2^ of 0% signifying no observed heterogeneity while a value > 30% signifying large heterogeneity [12]. We did not assess for publication bias as it is not indicated when there are less than 10 studies in a meta-analysis [13]. We used the weighted mean difference (WMD) for summary statistics as a measure of treatment effect to evaluate the comparison of SGLT2i and placebo. When the mean change from baseline to endpoint was not reported, the mean change was calculated by subtracting the mean at baseline from that at postintervention [13]. All statistical tests were carried out at a two-sided 5% level of significance using Review Manager 5.4 (Cochrane Collaboration, London, United Kingdom).

## 3. Results

### 3.1. Study Selection and Patient Characteristics

**Figure 1:**
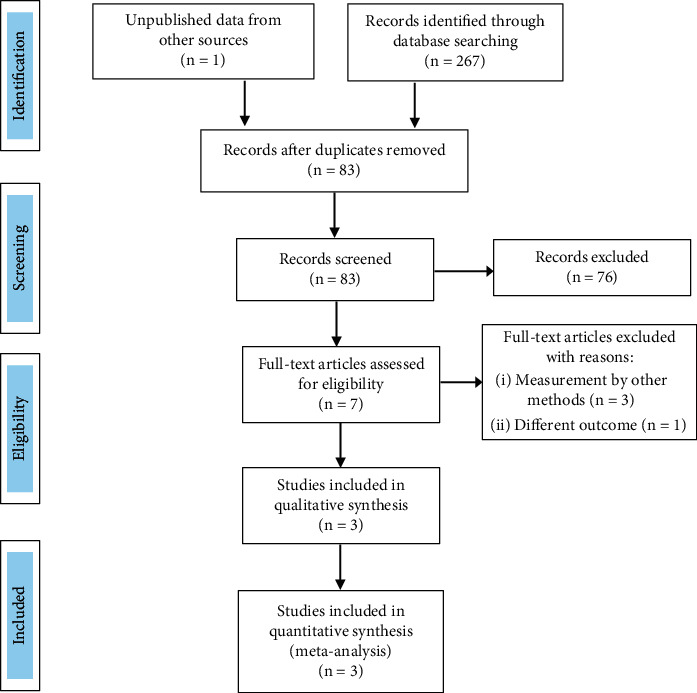
PRISMA flow chart.

The systematic search identified a total of 83 articles after the exclusion of duplicates (Figure 1). After screening and application of prespecified inclusion criteria, 3 studies (EMPIRE HF, SIMPLE, and CAMEO-DAPA) with a total of 145 patients (29% were female, 56.6% were in New York Heart Association functional Class II) were included in the meta-analysis [14–16]. Overall, there is a low risk of bias in the included studies in most domains except for some concerns due to missing data in a few patients randomized to a study arm but did not undergo right heart catheterization (Table 1). Patient characteristics in the included studies (two from Denmark and one from the United States) are summarized in Table 2. The included studies were randomized, double-blinded, placebo-controlled trials with overall no significant heterogeneity.

### 3.2. Impact of SGLT2i, Compared to Placebo, on Rest and Peak Exercise PCWP

**Figure 2:**
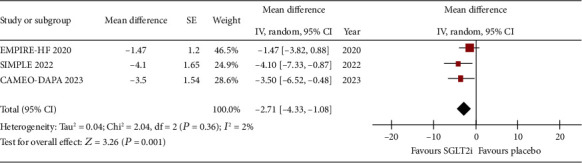
Forrest plot of weighted mean difference in rest PCWP (in mmHg) for patients on SGLT2i compared to placebo.

**Figure 3:**
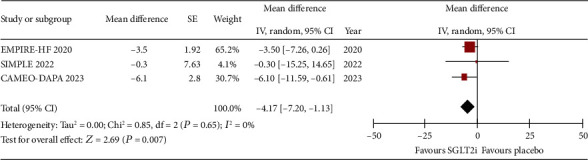
Forrest plot of weighted mean difference in peak exercise PCWP (in mmHg) for patients on SGLT2i compared to placebo.

The change in PCWP at rest and exercise was reported by the three studies. Figures 2 and 3, respectively, show that SGLT2i was associated with a reduction in PCWP at rest (WMD: −2.71, 95% CI: −4.33 to −1.08, *p* = 0.001, *I*^2^: 2%) and at peak exercise (WMD: −4.17, 95% CI: −7.2 to −1.13, *p* = 0.007, *I*^2^: 0%).

### 3.3. Impact of SGLT2i, Compared to Placebo, on Rest and Peak Exercise mPAP

**Figure 4:**
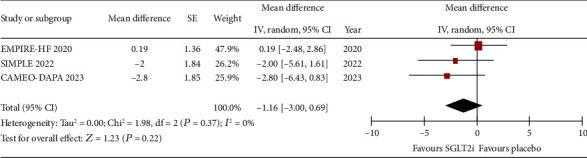
Forrest plot of weighted mean difference in rest mPAP (in mmHg) for patients on SGLT2i compared to placebo.

**Figure 5:**
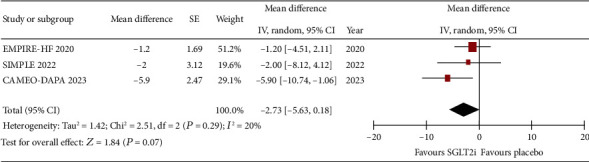
Forrest plot of weighted mean difference in peak exercise mPAP (in mmHg) for patients on SGLT2i compared to placebo.

The difference in pre- and postintervention invasive hemodynamic measurement showed that, compared to placebo, SGLT2is are not significantly associated with reduction in mPAP at rest (WMD: −1.16, 95% CI: −3.0 to 0.69, *p* = 0.22, *I*^2^: 0%) and peak exercise (WMD: −2.73, 95% CI: −5.63 to 0.18, *p* = 0.07; *I*^2^: 20%) (Figures 4 and 5, respectively).

## 4. Discussion

To the best of our knowledge, this is the first meta-analysis assessing invasive hemodynamic changes associated with SGLT2i. Based on our pooled analysis, compared to placebo, SGLT2i reduces PCWP at rest and further declines during peak exercise. SGLT2i was not statistically associated with a reduction in mPAP when compared to placebo. The result of this study offers mechanistic insights into the association of favorable hemodynamic effects underpinning the HF benefits of SGLT2i.

SGLT2is are one of the foundational “pillars” of HF management because of the evident morbidity and mortality benefits across diabetes status, LVEF, and a wide range of baseline kidney function [17]. However, the mechanisms of its benefit remain understudied with several overlapping and complementary pathways proposed, especially with respect to the effects on sodium excretion, volume status, and blood pressure [18]. Our study showed that SGLT2i significantly reduced PCWP at rest and at exercise. We hypothesized that the lowering effect of SGLT2i on the PCWP indirectly improves right ventricular function because it has been shown that a higher PCWP leads to increased pulsatile right ventricular load which contributes to right ventricular dysfunction [19]. The magnitude of PCWP reduction when compared to placebo increased during exercise implying that since exertional limitations are a cardinal manifestation of HF [20], the ability of SGLT2i to lower the filling pressure both at rest and even more at peak exercise may explain the lower rates of incident and hospitalized HF that has been attributed to this group of medications. Another clinical relevance of this finding is that since PCWP increases abnormally in patients with exercise phenotype of HF with preserved ejection fraction (HFpEF) [21, 22], we hypothesized that SGLT2i may play a beneficial role in improving exercise tolerance and functional capacity of this subset of patients because of its ability to blunt the steep increase in PCWP during exercise. This hypothesis is in keeping with the outcome of the DELIVER trial which showed that, across the range of LVEF, dapagliflozin improved physical limitations and quality of life across all domains of the Kansas City Cardiomyopathy Questionnaire [23]. Given that, of the three included studies, only CAMEO-DAPA had patients exclusively with HFpEF, further studies preferably done using extensive cardiopulmonary testing with invasive hemodynamic monitoring in patients with HFpEF will be needed, to make a definitive conclusion.

While our analysis identified a reduction in rest and peak exercise PCWP, the absolute magnitude of the decrease, compared to placebo, was below what we anticipated of a class of medication assumed to be acting as a “diuretic.” Though central hemodynamics does not linearly correlate with volume changes [24], based on the magnitude of reduction in PCWP by SGLT2i in our study, it is reasonable to assume that compared to conventional diuretics, volume depletion from SGLT2i may not overactivate the renin-angiotensin-aldosterone system like traditional diuretics, because SGLT2i induces more reduction in interstitial volume than intravascular volume [8, 25, 26]. Similarly, with the understanding that an increase in PCWP with exercise in patients with HF is closely linked to mortality [27], this is consistent with the robust cardiovascular mortality benefit seen in SGLT2i use in large cardiovascular outcome trials.

In our meta-analysis, SGLT2i was not significantly associated with a reduction in mPAP both at rest and peak exercise. This finding is consistent with the finding of the EMBRACE-HF trial where pulmonary pressure was monitored frequently via CardioMEMS. In that study, like our finding, the reduction in mPAP in the empagliflozin cohort was not statistically different compared to the placebo group [28]. Our result however contrasted with the findings of two single-arm observational studies by Mullens et al. and Kirschbaum et al. that showed that SGLT2i significantly reduced mPAP [29, 30]. Of note, while all the studies in this current study utilized RHC, mPAP was measured based on the reading of an implantable sensor device in the two observational studies. Also, the two studies have smaller sample sizes (9 and 17 patients, respectively), which are subject to cofounders being nonrandomized by design, and the single-arm nature of the studies limits the ability to detect if the reduction is statistically different compared to placebo [29, 30].

It is surprising that the significant reduction in PCWP noted in the patients in our analysis did not translate to a significant reduction in mPAP. We postulate that this may be due to the differences in the characteristics of the patient population as the SGLT2i cohort had higher baseline mPAP than the placebo group (average mPAP at rest in the SGLT2i group vs. placebo group in the EMPIRE-HF trial was 20 ± 6 mmHg vs. 17 ± 6 mmHg and in the SIMPLE trial was 22 ± 5 mmHg vs. 19 ± 5 mmHg). Further larger randomized trials in patients with baseline pulmonary hypertension are needed to conclusively explore the impact of the SGLT2i on pulmonary artery pressure changes.

In conclusion, SGLT2i significantly reduces rest and peak exercise PCWP but not mPAP in NYHA class II-III HF patients with ages > 18 years. The significant reduction in PCWP both at rest and at exercise associated with SGLT2i in this study may explain the mechanism behind how SGLT2i improves HF outcomes as elevated PCWP has been previously associated with dyspnea, exercise intolerance, reduction in quality of life, increased risk of HF hospitalization, and mortality. The result of this current study should be interpreted in the background of some limitations. It is a study-level meta-analysis, and we did not have access to individual patient data needed to estimate their possible interaction on the outcomes. Despite this limitation, our meta-analysis offers an evidence-based mechanistic insight into the association of favorable hemodynamic effects underpinning the HF benefits of SGLT2i. Noninvasive measurements like echocardiographic estimation of the ratio of early diastolic mitral inflow velocity to early diastolic mitral annulus velocity (*E*/*e*′) have been shown to not reliably reflect hemodynamic changes; hence, the utilization of right heart catheterization (the gold standard of central hemodynamics measurement) in all the studies in our meta-analysis is a notable strength of this study [31]. Future studies are needed to determine if the hemodynamic changes mediated by SGLT2i are sustained in the long term.

## Figures and Tables

**Table 1:** Risk of bias assessment (RoB2 tool).

**Domains**	**CAMEO-DAPA**	**EMPIRE-HF**	**SIMPLE**
Bias arising from the randomization process	Low risk	Low risk	Low risk
Bias due to deviations from intended interventions	Low risk	Low risk	Low risk
Bias due to missing outcome data	Low risk	Unclear risk	Unclear risk
Bias in measurement of the outcome	Low risk	Low risk	Low risk
Bias in selection of the reported result	Low risk	Low risk	Low risk

**Table 2:** Study and patient characteristics.

**Study**	**CAMEO-DAPA**		**EMPIRE-HF**		**SIMPLE**	
Country	USA		Denmark		Denmark	
Duration	24 weeks		12 weeks		13 weeks	
Intervention	Dapagliflozin 10 mg daily (*n* = 21)	Placebo (*n* = 17)	Empagliflozin 10 mg daily (*n* = 35)	Placebo (*n* = 35)	Empagliflozin 25 mg daily (*n* = 18)	Placebo (*n* = 19)
Age (years)	67 ± 9	67 ± 9	59 ± 8	56 ± 11	65 ± 9	63 ± 11
Female	14 (67%)	11 (65%)	3 (9%)	4 (11%)	7 (39%)	3 (16%)
White	35 (7.2%)	34.5 (5.5%)	35 (100%)	35 (100%)	NA	NA
BMI	35 ± 7.2	34.5 ± 5.7	29 ± 5	30 ± 6	32 ± 6	31 ± 6
NYHA II	7 (33%)	5 (29%)	27 (77%)	31 (89%)	8 (53%)	4 (25%)
NYHA III	14 (67%)	12 (71%)	8 (23%)	4 (11%)	0	1 (6%)
LVEF (%)	61 ± 6	63 ± 6	NA	NA	55 ± 8	59 ± 7
ACEi/ARB	6 (29%)	4 (24%)	25 (71%)	29 (83%)	17 (94%)	13(68%)
ARNI	—	—	9 (26%)	4 (11%)	NA	NA
Beta-blocker	6 (29%)	9 (53%)	32 (91%)	31 (89%)	9 (50%)	9 (48%)
MRA	7 (33%)	6 (35%)	22 (63%)	20 (57%)	1 (6%)	0
Diuretics	12 (57%)	12 (71%)	24 (69%)	26 (74%)	2 (11%)	4 (21%)

Abbreviations: ACEi: angiotensin-converting enzyme inhibitor; ARB: angiotensin receptor blocker; ARNI: angiotensin receptor/neprilysin inhibitor; BMI: body mass index; LVEF: left ventricular ejection fraction; MRA: mineralocorticoid receptor blocker; NYHA: New York Heart Association; USA: United States of America.

## Data Availability

The data/articles used to support the findings of this study are included within the article.
